# Uncovering kinesin dynamics in neurites with MINFLUX

**DOI:** 10.1038/s42003-024-06358-4

**Published:** 2024-05-29

**Authors:** Jan Otto Wirth, Eva-Maria Schentarra, Lukas Scheiderer, Victor Macarrón-Palacios, Miroslaw Tarnawski, Stefan W. Hell

**Affiliations:** 1 Department of Optical Nanoscopy, Max Planck Institute for Medical Research, 69120 Heidelberg, Germany; 2 Protein Expression and Characterization Facility, Max Planck Institute for Medical Research, 69120 Heidelberg, Germany; 3 Department of NanoBiophotonics, Max Planck Institute for Multidisciplinary Sciences, 37075 Göttingen, Germany

**Keywords:** Single-molecule biophysics, Biophysical methods

## Abstract

Neurons grow neurites of several tens of micrometers in length, necessitating active transport from the cell body by motor proteins. By tracking fluorophores as minimally invasive labels, MINFLUX is able to quantify the motion of those proteins with nanometer/millisecond resolution. Here we study the substeps of a truncated kinesin-1 mutant in primary rat hippocampal neurons, which have so far been mainly observed on polymerized microtubules deposited onto glass coverslips. A gentle fixation protocol largely maintains the structure and surface modifications of the microtubules in the cell. By analyzing the time between the substeps, we identify the ATP-binding state of kinesin-1 and observe the associated rotation of the kinesin-1 head in neurites. We also observed kinesin-1 switching microtubules mid-walk, highlighting the potential of MINFLUX to study the details of active cellular transport.

## Abstract (web summary)

A MINFLUX tracking study of a truncated kinesin-1 mutant in gently fixed primary rat hippocampal neurons revealed 8 nm substeps and a rotation of its head during the unbound state with nanometer/millisecond resolution

## Introduction

Kinesin-1 (herein referred to as kinesin) is a homodimeric motor protein of the kinesin superfamily known for taking distinct steps along single microtubule protofilaments. In a step, the motor domains (heads) move by 16 nm to the next free binding site. Kinesin has been studied extensively using fluorescence and scattering microscopy techniques^1–6^, the latter having revealed that each step of the head consists of two ~8 nm-sized substeps^6–9^. While those scattering-based techniques exploit the high signal available from a laser, they come at the cost of requiring labels that are by an order of magnitude larger than the head itself. This size discrepancy raises the question whether the substeps are altered, or caused, by steric hindrance or other factors^10,11^.

Interestingly, substeps of the head have recently been visualized using MINFLUX^12,13^, a method that requires substantially fewer detected fluorescence photons for localizing single fluorophores than its established camera-based counterparts. MINFLUX localizes by probing with an excitation laser beam featuring a minimal (zero) intensity point or line at the center. Iterative matching of this minimal intensity center with the position of the fluorophore substantially reduces the number of photon detections required for attaining a certain localization precision, typically by about 100-fold^14^. As a result, tracking single fluorophores with a spatio-temporal resolution of the order of nanometer/millisecond is readily attained. Harnessing the minimally impeding approach provided by direct fluorophore labeling, MINFLUX has recently been applied to study the conformational changes of kinesin walking on microtubules. An essential part of the cytoskeleton, microtubules are relatively stiff hollow polymers serving as tracks for motor proteins in the cell. It has been found that kinesin binds ATP between substeps while only one of the heads is bound to the polymer, i.e., during the one-head-bound (1HB) state, and that the heads undergo orientational changes during the substep.

However, all studies showing substeps—no matter if performed with small or large labels – have been conducted with microtubules polymerized in vitro and immobilized onto glass coverslips. Therefore, it remains uncertain if substeps do also occur on microtubules that were naturally assembled in cells, as they are decorated with microtubule-associated proteins (MAPs) and other biomolecules. Besides, tubulin may have undergone post-translational modifications. All these factors can act as roadblocks, forcing a motor protein to circumvent^15–17^ the obstacle on its path. The way, in which the cellular microtubules also affect the stepping behavior of kinesin remains unclear. Although a recent MINFLUX study of kinesin in cells observed substeps^18^, the observation was so scarce that the question remained whether the observed steps are rare by nature or just rarely observed due to limited spatio-temporal resolution or impediment by the label.

Using a truncated (residues 1-560), cysteine-light mutant of kinesin, we show that substeps occur frequently and regularly in fixed primary rat hippocampal neurons (rHPNs) at up to physiological ATP concentrations. Moreover, we find an inverse relationship between the walking speed and the duration of the unbound state, providing evidence that ATP is taken up while only one head is microtubule-bound. Finally, in line with observations on microtubules immobilized onto glass coverslips, we observe label position dependent asymmetries in substep sizes as well as sideward displacements of kinesin walking on microtubules in neurites.

## Results and discussion

Here we use gently fixed rHPNs, maintaining the native structure of the microtubules, as previously described^19^. Neurons are highly relevant in this context, as they feature long and highly branched neurites, such as axons and dendrites, which require active transport of cellular cargo. Using fixed neurons, we employed purified motor proteins labeled at the head by maleimide coupling and controlled the ATP concentration of the buffer.

**Fig. 1:**
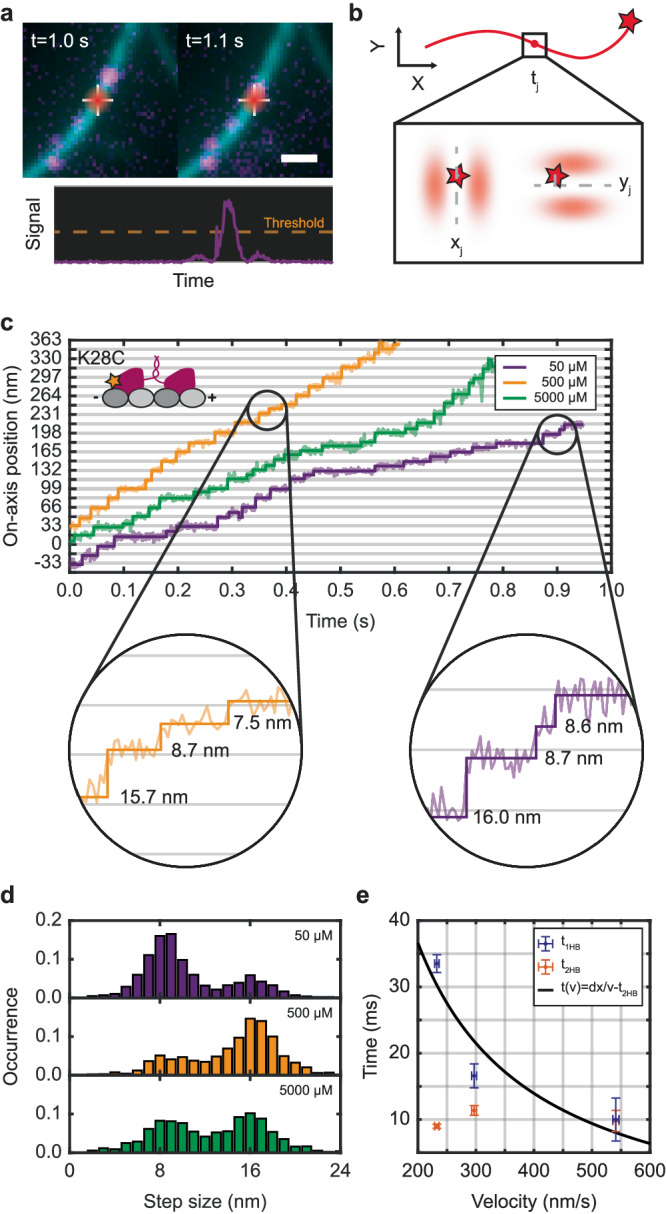
Visualization of substeps in primary rat hippocampal neurites. **a** Fluorophore-labeled kinesin motor walking into the stationary confocal volume (top) creating an increased signal and triggering a MINFLUX tracking measurement (bottom). **b** Schematic of the MINFLUX tracking process. For each time step t_j_ the x- and y-position of the minimum are updated to the newly estimated emitter position. **c** Exemplary on-axis position-time traces of construct K28C (inset) labeled at the back of the head domain recorded at 50 µM (purple), 500 µM (orange) and 5 mM (green) ATP concentration. The raw position data (semi-transparent lines) are overlaid with a step-fit (solid lines). Zoom-ins highlight ~16 nm regular steps and pairs of similar-sized ~8 nm substeps. **d** Population-normalized histograms of the measured step sizes recorded at the three ATP concentrations showing two populations of step sizes; 8 nm substeps and 16 nm regular steps (N_50µM_ = 1699, N_500µM_ = 1931, N_5mM_ = 1818). **e** Average duration of the one-head-bound (1HB) state between substeps (purple) and the two-head-bound (2HB) state (orange) plotted over the average velocity at 50 µM ($\bar{V}=232\pm 10$ nm s^−1^ s.e.m), 500 µM ($\bar{V}=541\pm 13$ nm s^−1^ s.e.m) and 5 mM ($\bar{V}=297\pm 11$ nm s^−1^ s.e.m). The solid black line shows a fit modeling the 1HB duration to velocity dependence by a simple rational function with parameters ${{{{{\rm{dx}}}}}}=9.1\pm 5.8\,{{{{{\rm{nm}}}}}}$ and ${{{{{{\rm{t}}}}}}}_{2{{{{{\rm{HB}}}}}}}=8.8\pm 19.4\,{{{{{\rm{ms}}}}}}$_._ Dwell times and velocity were averaged from in total 348 traces of 12 biological replicates. Error bars denote the standard deviation. Scale bar: 1 µm (**a**).

Since substeps have been shown on microtubules polymerized on coverslips for ATP concentrations ranging from 10 µM to 1 mM^13^, and virtually no substeps have been found in cells at 5 mM^15^, we measured at 50 µM, 500 µM and 5 mM thus covering the complete range from low-ATP up to physiological concentrations. The MINFLUX tracking routine was triggered by single motors walking into a stationary confocal volume on a neurite (Fig. 1a). The tracking routine consisted of repeated localizations in the x- and y-directions using line-shaped minima oriented along the x- and y-axis (Fig. 1b).

The first motor investigated was labeled at amino acid position 28 (construct K28C), which is located near the N-terminus at the back of the head with respect to the walking direction; see exemplary traces recorded at the three ATP concentrations (Fig. 1c). Traces were processed by an iterative step fit and substeps were identified by applying a Hidden-Markov-Model (HMM). This also allows discerning between the labeled head being microtubule-bound (bound state after 16 nm steps) or not (unbound state between paired substeps). The full ~16 nm steps between binding sites are clearly visible and so are the substeps of ~8 nm. The step size histograms disclose that the fraction of detected substeps depends on the ATP concentration, with most substeps being detected at 50 µM ATP and the least being detected at 500 µM (Fig. 1d). Relating the detected step size to the average walking speed of each trace reveals the fraction of substeps being velocity dependent, with higher walking speed resulting in fewer substeps detected (Supplementary Fig. S1). This observation is supported by evaluating the duration of the unbound state in-between paired substeps and the duration of the bound state in-between two full steps. From the histograms of these durations the average durations t_1HB_ and t_2HB_ that kinesin spends in a one-head-bound (1HB) state and a two-head-bound (2HB) state are extracted assuming equal kinetics of both heads (Supplementary Fig. S2). While no strong dependence on the walking speed can be observed for the 2HB state duration, the duration of the 1HB state monotonously decreases with increasing walking speed (Fig. 1e). Furthermore, since the walking speed is proportional to the ATP turnovers and thus the ATP binding rate, we find that ATP binding occurs during the 1HB state in the neurites as well.

Also, the walking speed does not saturate with increasing ATP levels, as usually described in the literature^2,20,21^. We ascertained the decrease in walking speed at 5 mM ATP using fluorescence widefield microscopy, matching the walking speeds observed with MINFLUX (Supplementary Fig. S3).

**Fig. 2:**
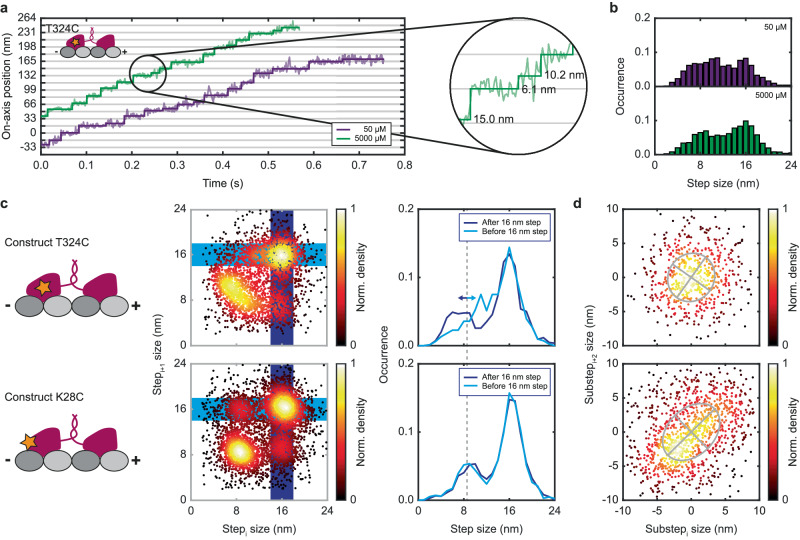
Comparison of the stepping behavior for constructs labeled at different amino acids. **a** Exemplary on-axis position-time traces of construct T324C (inset) labeled at the center-right of the head domain recorded at 50 µM (purple) and 5 mM (green) ATP concentration. The raw position data (semi-transparent lines) are overlaid with a step-fit (solid lines). The zoom-in highlights 16 nm regular steps and unequally-sized substeps. **b** Population-normalized histograms of measured step sizes recorded at the two ATP concentrations showing step sizes of 16 nm and substeps between 6 nm and 11 nm (N_50µM_ = 1625, N_5mM_ = 1592). **c** Comparison of the on-axis stepping behavior between construct T324C and K28C (constructs depicted on the left). The sequence of step sizes is displayed by a bivariate density scatter plot (middle). The step sizes recorded after a regular step (16 nm) are underlaid in dark blue, those before a regular step in light blue. The projections along the short axis of these boxes are shown as plots (right) showing similar substep sizes for construct K28C and varying substep sizes for construct T324C. The total number of steps for construct T324C and K28C are given by N_T324C_ = 3301 and N_K28C_ = 5519, respectively. **d** Comparison of the off-axis stepping behavior. Bivariate density scatter plots of the off-axis displacement for all subsequent bound to unbound and unbound to bound substeps together with the corresponding ellipses from the eigenvalues and eigenvectors of the covariance matrices (N_T324C_ = 590, N_K28C_ = 1055). Data for construct T324C is taken from 216 traces of 12 biological replicates (for statistics on construct K28C refer to Fig. 1).

Next, we switched to a motor labeled at amino acid position 324 (construct T324C). The label is designed to be close to the neck linker. To compare the stepping behavior to that of construct K28C, we recorded traces at 50 μM and also at 5 mM ATP concentration in order to maximize the number of detected substeps (Fig. 2a). Both 16 nm-sized full steps as well as many substeps could clearly be resolved.

However, the histogram of detected step sizes does not show a clear separation between full steps and substeps due to a rather broad substep distribution (Fig. 2b). To achieve an unbiased interpretation of this observation we plotted the size of consecutive steps against each other in a bivariate density scatter plot (Fig. 2c middle). The data clusters into four different regions centered around (16 nm, 16 nm), (16 nm, 8 nm), (8 nm, 8 nm) and (8 nm, 16 nm) with the greatest density at (16 nm. 16 nm) and (8 nm, 8 nm). As mentioned previously, we assume the labeled head to be microtubule-bound after a full step and unbound in-between paired substeps. Therefore substeps after a full step (16 nm, 8 nm) describe transitions from the bound to the unbound state which are paired with a substep before a full step (8 nm, 16 nm). Comparing the distributions of both constructs shows that, while the pairs of substeps of construct K28C uniformly spread around 8 nm, those of construct T324C are found to be in a range of 6 – 10 nm (combining to 16 nm) with the first substep being usually smaller than the second (Fig. 2c right).

We evaluated the sidewards displacement during the unbound state by plotting the off-axis step size of consecutive bound-to-unbound and unbound-to-bound transitions against each other in a bivariate density scatter plot (Fig. 2d). Here we reach the limit of our localization precision (4-5 nm standard deviation for single localizations) as the sidewards displacement is typically on the same scale or smaller. However, while no clear correlation can be observed for the off-axis step size of construct T324C, a small correlation can be observed for construct K28C, consistent with the previously observed rotation of the head during the unbound state^13^.

**Fig. 3:**
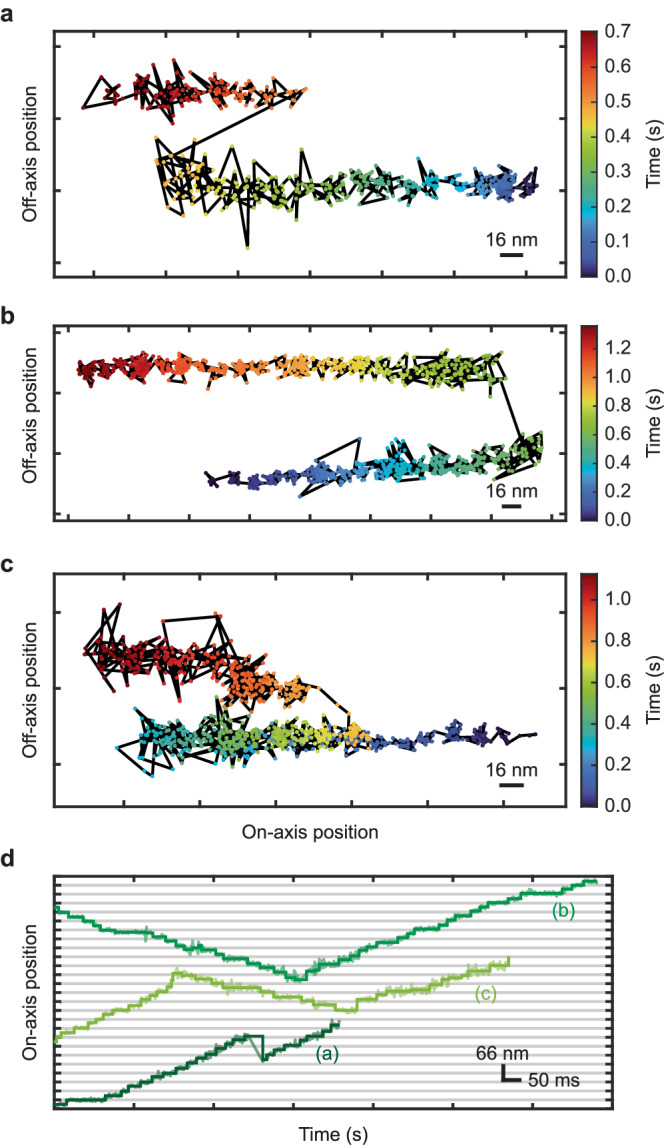
Kinesin switches microtubules and changes walking direction in the cellular context. **a–c** Exemplary 2D position traces of construct K28C color-coded for time, all displaying an off-axis displacement larger than the ~25 nm diameter of a microtubule, indicating mid-walk microtubule switching. Individual localizations (points) are connected by black lines. The traces displayed in (**a, b**) show an off-axis displacement of ~60 nm within 15–50 ms. While trace (**a**) continues progressing into the same direction, trace (**b**) switches to walking into roughly the opposite direction. The trace shown in (**c**) exhibits microtubule switching with ~60 nm off-axis displacement within less than 5 ms, accompanied by a previous change in walking direction without off-axis displacement (indicated by the time color-coding). **d** Position-time traces of the on-axis movement of the traces shown in (**a**–**c**). The raw data (semi-transparent lines) are overlaid by the step fit (solid lines).

Lastly, we observed kinesin switching microtubules mid-walk (Fig. 3a–d). Specifically, we saw kinesin motors displaying abrupt changes to their off-axis position and occasionally reversing their walking direction (Fig. 3b, c). As kinesin can only walk towards the plus- end of microtubules, no retrograde motors are present in the sample and the typical rate of recording traces was <1*s*^−1^, this phenomenon can most likely be explained by a microtubule switch. The observed switching occurred on small distances as well as between up to 80-nm-spaced microtubules, much larger than the typical 8 nm spacing between the heads. One of the examples (Fig. 3c), even exhibits two switches (at 0.3 s and 0.8 s) within a single trace. As the data represents a 2D-projection of the actual 3D movement, we assume the first switching was to a microtubule separated in the z-direction.

In conclusion, MINFLUX directly visualizes kinesin substeps on microtubules of lightly fixed neurites in a cellular context. While we validated the stepping characteristics that were recently reported for microtubules polymerized in vitro and immobilized onto glass coverslips in neurites, the higher microtubule density in axons and dendrites allowed us to observe additional effects, such as microtubule switching. The recorded changes in the walking pattern of motors labeled at different positions highlight the importance of pursuing adequate and possibly multiple labeling strategies to minimize the risk of drawing conclusion influenced by the position and flexibility of the fluorophore. In any case, MINFLUX tracks the largely unimpeded motion of motor proteins in unprecedented detail, enabling the unraveling of protein dynamics and conformational changes directly in a cell.

## Materials and methods

### Primary rat hippocampal neuron culture

Primary rat hippocampal neurons (rHPNs) were prepared from postnatal P0-P2 Wistar rats (Janvier Labs, Le Genest-Saint-Isle, France) of either sex with no genetic modification as previously described^22^. We have complied with all relevant ethical regulations for animal use. Animals were sacrificed according to the guidelines established by the German Animal Welfare Act (Tierschutzgesetz der Bundesrepublik Deutschland, TierSchG) and the Animal Welfare Laboratory Animal Ordinate (Tierschutz-Versuchstierverordnung: TierSchVersV), following all regulations, conducted and documented under supervision of animal welfare officers of Max Planck Institute for Medical Research (MPImf) (permit number assigned by the MPImF: MPI/T-35/18). According to the TierSchG and the Tierschutzversuchstierverordnung no ethical approval from the ethics committee is required for the procedure of sacrificing rodents for subsequent extraction of tissues, as performed in this study. Briefly, hippocampi were collected and dissected all together from the same litter, digested with 0.25% trypsin (Thermo Fisher Scientific, Waltham, MA, USA) for 20 min at 37 °C and subsequently dissociated and maintained in cell culture medium (Neurobasal™ supplemented with 2% B-27, 1% GlutaMAX and 1% penicillin/streptomycin; all from Gibco, Thermo Fisher Scientific). Dissociated cells were plated at a density of 60,000 cells/well in 12-well plates on 18 mm coverslips pre-coated with 0.1 mg ml^−1^ poly-ornithine (Sigma-Aldrich/Merck, Darmstadt, Germany) and 1 μg ml^−1^ laminin (Corning, New York, NW, USA) in culture medium. Medium was exchanged after attachment of the cells (approximately 2 h) and neurons were maintained at 37 °C in a humidified incubator (95% rH) with 5% CO_2_ until 11 - 21 days in vitro (DIV).

### Constructs

Truncated human kinesin-1 (residues 1-560 with a C-terminal His-tag) with all solvent-exposed cysteines mutated to alanine or serine (C7S, C65A, C168A, C174S, C294A, C330S, C421A) and containing a unique cysteine residue for labeling at position T324C was expressed using plasmid K560CLM T324C (obtained from Addgene, #24460). A ‘cysteine-light’ truncated human kinesin-1 for labeling at K28C position (K560CLM K28C) was generated using QuikChange II (Agilent) site-directed mutagenesis method with CLM RP HTR plasmid obtained from Addgene (#24430) and used as a template. The construct was verified by Sanger sequencing (Eurofins).

### Protein purification and labeling

The vectors were transformed into E. coli BL21 CodonPlus(DE3)-RIL (Agilent). Cells were grown at 37 °C in LB medium supplemented with ampicillin (100 μg ml^−1^) and chloramphenicol (30 μg ml^−1^). At an optical density at 600 nm of 0.8-1.0, cells were transferred to 18 °C and expression was induced with 0.1 mM isopropyl β-D-1-thiogalactopyranoside (IPTG). Cells were harvested after overnight expression by centrifugation, frozen in liquid nitrogen and stored at -80 °C until further use. All subsequent purification steps were done at 4 °C.

The cell pellets were resuspended in lysis buffer (50 mM NaH2PO4 pH 8.0, 250 mM NaCl, 20 mM imidazole, 2 mM MgCl2) supplemented with cOmplete EDTA-free protease inhibitors (Roche), 20 μg ml^−1^ DNaseI, 10 mM β-mercaptoethanol, 1 mM ATP. The cells were lysed using a microfluidizer (Microfluidics) operated at a pressure of 0.9 MPa and the lysates were clarified by centrifugation at 47,850 g for 1 hour at 4 °C. The cleared supernatants were loaded onto a HisTrap FF 5 ml (Cytiva) column pre-equilibrated with lysis buffer. The column was washed with 50 mM NaH2PO4 pH 6.0, 250 mM NaCl, 20 mM imidazole, 1 mM MgCl2, 10 mM β-mercaptoethanol, 0.1 mM ATP and the protein was eluted with 50 mM NaH2PO4 pH 7.2, 250 mM NaCl, 500 mM imidazole, 1 mM MgCl2, 10 mM β-mercaptoethanol, 0.1 mM ATP.

Fractions containing kinesin were 5-fold diluted with buffer A (25 mM PIPES pH 6.8, 2 mM MgCl2, 1 mM EGTA, 0.2 mM TCEP, 0.1 mM ATP) before loading onto a HiTrapQ FF 5 ml (Cytiva) column pre-equilibrated with buffer A containing 100 mM NaCl. The column was washed with the same buffer and the protein was subsequently eluted using linear gradient of 100-1000 mM NaCl in buffer A. The peak fractions containing kinesin were concentrated using Amicon Ultra (Merck Millipore) centrifugal units and further purified by gel filtration on a HiLoad 16/600 Superdex 200 pg (Cytiva) column equilibrated with buffer B (25 mM PIPES pH 6.8, 300 mM NaCl, 2 mM MgCl2, 1 mM EGTA, 0.2 mM TCEP, 0.1 mM ATP) to further improve the sample quality. Selected fractions containing kinesin were finally combined and concentrated, supplemented with 10% (w/v) sucrose, aliquoted, frozen in liquid nitrogen and stored at –80 °C. Purified proteins were analyzed by ESI-MS.

Exposed solvent cysteines were labeled with ATTO 647 N maleimide (AD 647N-41, ATTO-TEC) over night at 4 °C and excess dye was removed by size-exclusion chromatography (PD MiniTrap G-25, 28-9180-07, Cytiva). The degree of labeling was determined wit UV-Vis spectroscopy (DS-11+ Spectrophotometer, DeNovix) and mass spectrometry (ESI, maXis II ETD, Bruker) and aliquots of purified labeled kinesin were flash frozen in PEM80 (80 mM PIPES, 0.5 mM EGTA, 2 mM MgCl2 set to pH 7,40) supplemented with 10% (w/v) sucrose and stored at −80 °C until use.

### Cell fixation

To prepare neurons for tracking, the cytoplasm was extracted by applying prewarmed 37 °C extraction buffer (1 M sucrose and 0.15% (v/v) Triton-X-100 (AppliChem GmbH) in BRB80: 80 mM PIPES, 1 mM EGTA, 1 mM MgCl_2_ set to pH 6.80) for 1 min. Next, the buffer was exchanged for prewarmed fixation buffer (0.5 M sucrose, 0.075% (v/v) Triton-X-100 and 1% (v/v) paraformaldehyde (PFA, Carl Roth) in BRB80) for 1 min. Subsequently the solution was exchanged for prewarmed washing buffer (1 µM paclitaxel (Focus Biomolecules) in BRB80) and incubated for 1 min. The washing step was repeated three times. Fixed microtubules were stained with BioTracker™ 488 Green Microtubule Cytoskeleton Dye (SCT142, Sigma-Aldrich/Merck; 1:1000 in BRB80) and incubated for at least 15 min at 37 °C. The staining solution was removed and the cells were blocked for 3 min with blocking buffer (2 mM ascorbic acid (BioVision Inc), 0.5 mg ml^−1^ casein (VWR Chemicals), 538 μg ml^−1^ catalase, 1 mM 1,4-dithiothreitol (DTT, Carl Roth), 41.7 μg ml^−1^ glucose oxidase, 1.7% (w/v) glucose, 1 µM paclitaxel, 2 mM methyl viologen and 50 µM/500 µM/5 mM ATP; (all from Sigma-Aldrich/Merck unless stated otherwise) in BRB80). Finally coverslips with fixed cells were mounted on cavity slides (1320002, Paul Marienfeld GmbH & Co.KG, Lauda Königshofen, Germany) containing approximately 150 μL imaging buffer (2 mM ascorbic acid, 0.2 μg ml^−1^ casein, 538 μg ml^−1^ catalase, 1 mM DTT, 41.7 μg ml^−1^ glucose oxidase, 1.7% (w/v) glucose, 1 µM paclitaxel, 2 mM methyl viologen and 50 µM/500 µM/5 mM ATP in BRB80) supplemented with 4-8 nM kinesin and edges were sealed with picodent twin-sil speed 22 (picodent Dental-Produktions- und Vertriebs GmbH) and cured for 5 min at 37 °C.

### MINFLUX tracking routine

Before recording MINFLUX position traces of kinesin, suitable microtubule regions were selected by performing 5×5 μm^2^ confocal xy-scans of the 488 nm laser (pixel size 50 nm). Typically, thin and straight bundles were selected, with minimal background and devoid of overlapping processes. Up to 30 pixels were manually selected in a zig-zag arrangement over the width of a microtubule bundle and sequentially addressed by the galvo-scanner with 10 ms exposures of the 642 nm laser. Once the detected photon rate exceeded a predefined threshold ranging from 6 to 10 kHz, depending on the background of an individual sample, the MINFLUX tracking routine was triggered. During the initial zoom-in phase of the routine, a series of three MINFLUX steps with progressively decreasing L were performed, probing the excitation intensity minimum around the latest estimated molecule localization to positions $[-L/2,0,L/2]$ in both dimensions. Following each cycle, the molecule’s position estimate *x* was updated based on the recorded photons according to

1 $${x}_{{new}}={x}_{{old}}+\frac{L}{4}\frac{{n}_{-}-{n}_{+}}{{n}_{+}+{n}_{-}-2{n}_{0}}$$

with $[{n}_{-},{n}_{0},{n}_{+}]$ being the photons recorded at $[-L/2,0,\,L/2]$ respectively. Localizations are considered failed if either less than five photons are collected or if $2\left({n}_{+}+{n}_{-}-2{n}_{0}\right) < \left|{n}_{-}-{n}_{+}\right|$ which limits the allowed corrections to *L / 2*. Repeated localizations with the same *L* ensure that no motor is missed the localization process due to a single failed localization. As the first three steps only ensure the centering of the dye, most repeats are performed in the last step. At this step *L* was set to 50 nm for all measurements. For each dimension a localization consists of three exposures with a set exposure time of 200 μs. Considering an additional dead time of 5 μs for switching between exposures and 500 ps for recalculating the updated molecule position, a single two-dimensional (2D) localization process required 1231 μs. An overview over the *L*-values, laser powers and repeats is given in Supplementary Table S1.

### TIRF/Widefield microscopy

Kinesin velocity measurements were performed on a custom widefield setup built around an Olympus IX83 microscope body (Olympus Corp., Tokyo, Japan) providing a motorized stage and a piezo Z-stage. The system is equipped with two excitation laser lines at wavelength 488 nm (Oxxius, Lannion, France) and 640 nm (Cobolt AB, Solda, Sweden). For image acquisition, the excitation laser was focused in the back focal plane of the 1.49 NA 100× oil objective lens (UAPON100XOTIRF, Olympus Corp.) controlled by an acousto optical tunable filter (AOTF, AA OPTO-ELECTRONIC, Orsay, France) and the emitted light was passed via a quad-band emission filter (F66-887, AHF analysentechnik AG, Tübingen, Germany) for separation of excitation and emission bands, respectively. Total internal reflection fluorescence (TIRF, Fish, 2009) illumination mode was achieved by moving the incident laser beam via the motorized stage to the outer part of the pupil plane. Emitted photons from the sample were captured by an iXon 897 EMCCD camera (Andor Technology, Belfast, UK). Active z-stabilization was provided based on the Olympus focus stabilization system (Confocus) and the microscope was operated by a custom-written LabView (National Instruments Corp., Austin, TX, USA) program.

Frame stacks were recorded at 50 μM, 500 μM and 5000 μM ATP concentration in 3 biological samples each. For each condition, 4 cells were imaged and time lapse acquisitions of 200 time points with an exposure time of 100 ms were captured. Moving kinesins (construct K28C) labeled with ATTO 647 N on fixed neuronal microtubules were recorded with the 642 nm laser set to 5 mW laser power. (176 W cm^−2^ focal intensity) Microtubules were imaged using the 480 nm laser at 1 mW laser power (353 W cm^−2^ focal intensity). Frame series were analyzed in Fiji^23^ determining the run length along microtubules for individual motors. Velocities were calculated from determined run-lengths over time.

### Data analysis

MINFLUX position traces of kinesin were screened and selected in LabView 2019 based on a sufficient number of detected photons for a successful localization, as well as them displaying a clear stepping behavior and a good average SBR. Those traces were post-processed and analysed using dedicated Matlab scripts. First, the start and end of each trace are manually selected. Then, using a so-called sliding curvature estimator

2 $${x}_{{SCE}}(t)=\frac{L}{4}\frac{{n}_{-}(t)-{n}_{+}(t)}{1/T{\int }_{t{\prime} -T/2}^{t{\prime} +T/2}{n}_{+}(t{\prime} )+{n}_{-}(t{\prime} )-2{n}_{0}(t{\prime} ){dt}{\prime} }$$

the position estimate was refined by averaging the curvature of the excitation pattern at each time t in a sliding time interval of around *T* = 20 ms. Typically, kinesin does not move within this time interval, thus validating to assume the curvature constant. By doing so, noise on the position estimate can be reduced, improving the localization precision without loss of temporal resolution. Next, the main movement of the trace is aligned with the x-axis. This so-called on-axis x-position is subjected to the step fit function described in^13^, which is based on an iterative change point detection^24^. The step function is additionally filtered by a moving median of width 9 (to remove steps caused by spikes in the data) and in a single round, steps below 2 nm are removed. Note that removing steps changes the plateau levels and thus affects the following or previous step and can cause them to become less than 2 nm. The change points of the on-axis step fit are then used to create the step fit for the off-axis position. From the step fits, the step sizes as well as the dwell time between steps are extracted.

In order to extract the dwell times between substeps and regular steps, as well as to correlate the off-axis displacement between substeps, the sequence of steps from single traces are analysed using a Hidden Markow model^13^. The model identifies the most likely sequence of the labeled head being bound or unbound based on the assumptions that regular steps are 16 nm-sized and that substeps occur in pairs each being around 8 nm in size.

Additionally, the average localization precision and photon counts within each plateau are extracted as well as the run length and run time, from which the velocity can be calculated. All this information is saved in an excel sheet for pooled processing.

To further evaluate the average residence time kinesin spends in the one-head-bound (1HB) and two-head-bound (2HB) state the dwell times of the bound and unbound state pooled from all traces recorded with the same construct and ATP concentration are fitted simultaneously using the model derived in^13^.

3 $$\begin{array}{c}{p}_{U}\left(t\right)=\frac{{e}^{-\frac{t}{{\tau }_{1{HB}}}}}{{\tau }_{1{HB}}}\\ {p}_{B}\left(t\right)=\frac{1}{{\tau }_{2{HB}}\left({\tau }_{2{HB}}-{\tau }_{1{HB}}\right)}\left(\tau {e}^{-\frac{\tau }{{\tau }_{2{HB}}}}-\frac{{\tau }_{1{HB}}{\tau }_{2{HB}}}{{\tau }_{2{HB}}-{\tau }_{1{HB}}}\left({e}^{-\frac{\tau }{{\tau }_{2{HB}}}}-{e}^{-\frac{\tau }{{\tau }_{1{HB}}}}\right)\right)\end{array}$$

### Data representation

Traces illustrate the post-processed MINFLUX tracking position either over time together with their corresponding step function.

Histograms show the counts per individual bin normalized to the size of the population. Single datasets are displayed as bars and multiple datasets in a single plot as lines.

Scatter plots present either a sequence of step sizes, or xy-coordinates of a trace. For a sequence of step sizes each point is color-coded according to the number of points within a 4 nm radius. For xy-traces each point is color-coded for time.

Violin plots represent the probability density of values occurring in a data set. The probabilities are retrieved by the *ksdensity* function in matlab. All violins are scaled identically making all probabilities comparable.

### Statistics and reproducibility

A total of 348 MINFLUX traces of construct K28C were recorded, dividing into 125 traces (6 biological replicates) at 50 μM, 118 traces (3 biological replicates) at 500 μM and 105 traces (3 biological replicates) at 5 mM ATP concentration. A total of 216 MINFLUX traces of construct T324C were recorded, dividing into 114 traces (6 biological replicates) at 50 μM, and 102 traces (6 biological replicates) at 5 mM ATP concentration. For the widefield tracking three biological replicates were recorded for each ATP concentration. A total of 20 traces were evaluated per ATP concentration and biological replicate. Biological replicates refer to independently prepared samples.

### Reporting summary

Further information on research design is available in the Nature Portfolio Reporting Summary linked to this article.

### Supplementary information

Supplementary Information

Reporting Summary

## Data Availability

The numerical source data (raw data) behind the graphs shown in this study is available at 10.5281/zenodo.10718784^25^. All other data are available from the corresponding author (or other sources, as applicable) on reasonable request.

## Code availability

A software package of custom-written Matlab (2022b) code is available at 10.5281/zenodo.10718784^25^.
