# Supplementary material for: Uncovering kinesin dynamics in neurites with MINFLUX
Source: Commun Biol. 2024 May 29;7:661. doi: 10.1038/s42003-024-06358-4 (PMC11136979; doi:10.1038/s42003-024-06358-4)
Supplement: Supplementary file 1 — Supplementary Information [file 42003_2024_6358_MOESM1_ESM.pdf]

Supplementary Information for

Uncovering kinesin dynamics in neurites with MINFLUX

Jan Otto Wirth[1†], Eva-Maria Schentarra[1†], Lukas Scheiderer[1], Victor Macarrón-Palacios[1], Miroslaw Tarnawski[2], Stefan W. Hell[1,3]*

[1] Department of Optical Nanoscopy, Max Planck Institute for Medical Research, 69120 Heidelberg, Germany

[2] Protein Expression and Characterization Facility, Max Planck Institute for Medical Research, 69120 Heidelberg, Germany

[3] Department of NanoBiophotonics, Max Planck Institute for Multidisciplinary Sciences, 37075 Göttingen, Germany

[†] These authors contributed equally

* Corresponding author: shell@gwdg.de

Content

1. Supplementary Information 2
2. Supplementary Tables 5

1. Supplementary Information

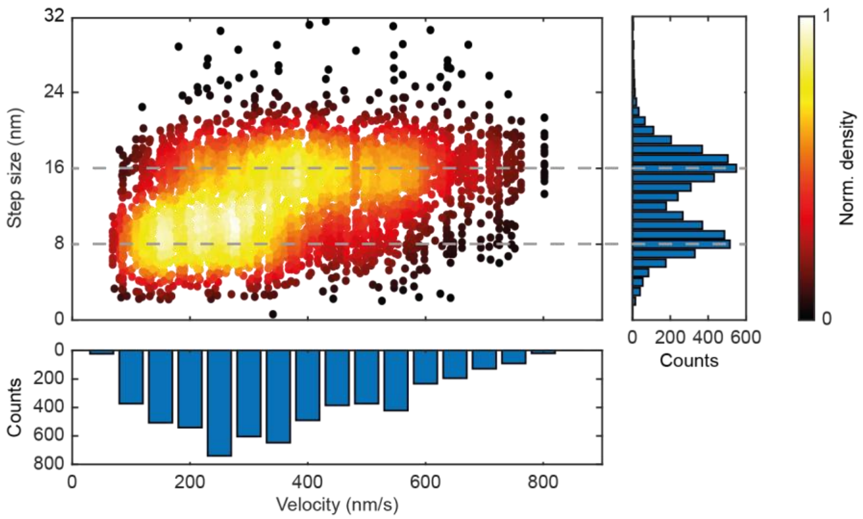

Supplementary Figure S1. Apparent step sizes vary with motor velocity. (Top left) Bivariate density scatter plot of recorded step sizes over the average velocity of the corresponding trace. The plot pools data recorded at 50 µM, 500 µM and 5 mM ATP concentration. The color coding denotes the number of points within 4 nm step sizes and 50 nm/s velocity. Step sizes of 8 nm and 16 nm are highlighted by grey dashed lines. (Top right) Histogram of the step sizes shown in the scatter plot. (Bottom) Histogram of the velocities shown in the scatter plot. The y axis is inversed. All data shown was recorded with construct K28C.

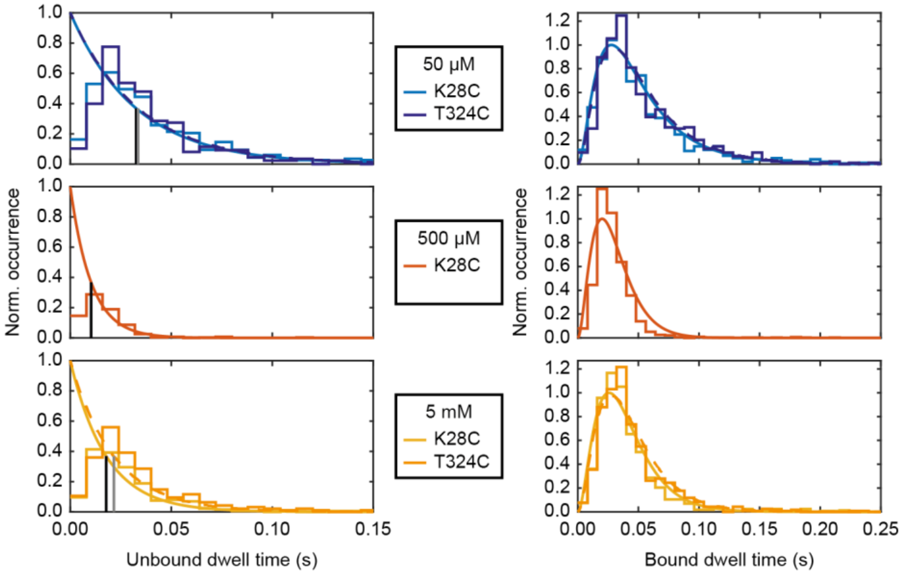

Supplementary Figure S2. Histogram of dwell times in the bound and unbound state. Stair plots of the dwell time histogram counts in the unbound state (left) and bound state (right) together with the fits according to a single exponential decay (unbound) and a convolution of three exponential decays with two rate constants. The convolution of three rate constant comprises two times the dwell time of the two-head bound state (labeled head leading and labeled head trailing) and once the dwell time of the one-head bound state (labeled head bound). The data is split by the employed ATP concentration (top 50 µM, middle 500 µM, bottom 5 mM) and constructs (K28C light blue and yellow, T324C dark blue and orange). For visibility, the fits for construct T324C are shown as dashed lines.

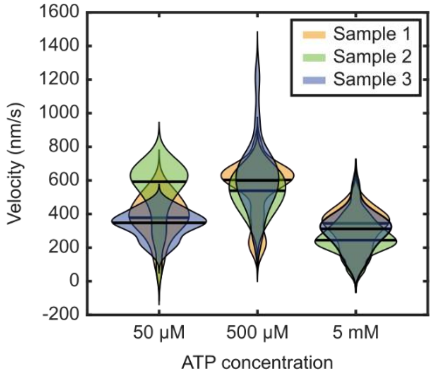

Supplementary Figure S3. Activity control for construct K28C. Violin plots of the motor velocity measured at 50 µM, 500 µM and 5 mM ATP concentration using a widefield fluorescence microscope. The width of the violin denotes the probability of a certain velocity and the black line the median. For each sample and ATP concentration, the velocity of 20 traces was recorded.

2. Supplementary Tables

Supplementary Table S1. MINFLUX tracking parameters. L-values, laser power (as measured going into the galvo scanner) and repeats used in the different MINFLUX steps. In each step a localization lasts 1231 µs.

Step	L (nm)	Laser power (µW)	repeats
1	240	6	3
2	120	24	5
3	70	70	10
4	50	180	1482
